# An exploratory study on needs for clinical research training: data from Chinese hospitals

**DOI:** 10.1186/s12909-021-02993-1

**Published:** 2021-11-02

**Authors:** Wei Liu, Wei Huang, Chang Liu, Pei Li, Jing Chen

**Affiliations:** 1 National Clinical Research Center for Aging and Medicine, Huashan Hospital, Fudan University, 12 Middle Wulumuqi Road, Jingan District, 200040 Shanghai, China; 2 Jiahui Medical Research & Education Group, 689 Guiping Road, Xuhui District, 200233 Shanghai, China

**Keywords:** Clinical research, Needs assessment, Physician-scientist

## Abstract

**Objective:**

Through preliminary investigation of needs for clinical research training, to provide some initial evidence for design and operation of clinical research training programs in China.

**Methods:**

An online questionnaire containing 23 questions about demographics, current practice of clinical research and needs for clinical research training was formulated to collect data from physicians and researchers in hospital. Convenience sampling was adopted.

**Results:**

A total of 600 valid questionnaires were collected, including 507 from physicians, 58 from full-time clinical researchers and 35 from full-time basic medical researchers. Results showed that 14 % of the participants never participated in any clinical research, difficulties in data statistics were reported by more than 70 % of participants, and over 50 % reported training needs for cohort and biobank construction, data management and clinical study design. As to the training form, a combined on-line lecture with off-line communication is preferred. As for the content of the training program, strategies for literature reading and grant writing are highly demanded.

**Conclusions:**

In China, even physicians in top hospitals tend to encounter various difficulties when conducting clinical research. This study highlights the importance of systematic training for future physician scientists in China, with a particular focus on data statistics, cohort and biobank construction and data management.

**Supplementary Information:**

The online version contains supplementary material available at 10.1186/s12909-021-02993-1.

## Background

With a rapidly ageing population, accompanied by rapid developments in disease diagnosis, treatment methods and disease spectrum, the importance of clinical research is becoming increasingly prominent in China [1]. China’s clinical research yield still lags behind the international research community on the fronts of both quantity and quality, as reflected by the total number of clinical trials, the number of phase I clinical trials, international multi-center clinical trials, and quality clinical research articles [2]. In addition, inadequate medical apparatus and instrument research and development capacity [3], few diagnosis and treatment guidelines and recommendations based on Chinese population, are also serious challenges for us [4].

With the establishment of national clinical research centers, the rollout of national clinical research programs, and the founding of clinical research units in top medical schools and hospitals in China, clinical research has been in the limelight since the “13th Five-Year Plan” period [5–7]. Meanwhile, both of the number of physicians and researchers involved in clinical studies and the funding for clinical research are on the rise. Concurrently, with the increase of novel knowledge and new technologies, emerging therapies are becoming more personalized, and the practice of multi-discipline treatment has become commonplace. Therefore, in order to take full advantage of all the above-mentioned medical resources, sufficient clinical research training is in urgent need [8, 9]. Physician-scientists, usually as the principal investigators of clinical research, need to focus more on team management, aside from the implementation of specific procedures [10]. Physicians who do not engage in clinical research still need to read and understand the clinical research conducted by others so as to stay informed of latest developments in clinical practice. Physicians are in urgent need of help with designing and conducting clinical research to improve clinical research quality [11–13].

Currently, the institutions the authors work for are jointly planning a clinical research training program. This study among Chinese physicians, clinical researchers, and basic medical researchers in hospitals was conducted to collect data and provide preliminary analysis on their needs for clinical research training. It is hoped this paper could provide initial evidence for the designing and operating of clinical research training programs.

## Methods

### Questionnaire construction

The questionnaire used in this study is constructed based on two researches. The first one is the *Comprehensive Evaluation Kit* developed by National Health Commission, and it is used to evaluate the performance of top-level hospitals in China based on the data submitted to the National Health Commission annually [14]. In this *Comprehensive Evaluation Kit*, clinical research training for physicians and researchers is a key dimension. The second one is a study aimed at analyzing physicians’ experience with and attitudes towards clinical trials [15]. The questionnaire contains 23 questions about demographics, current practice of clinical research and need for related training, in which question No. 9 to No. 16 is from *Comprehensive Evaluation Kit* and No. 17 to No. 23 is from the second study. No latent variable was identified or measured in this study. The questionnaire was designed and sent via an online survey tool which can be visited at https://www.wjx.cn/. The questionnaire is free for this study.

### Participant recruitment

The study intended to enroll physicians and researchers who work at hospitals, with or without experience of clinical research. The questionnaire link was sent to 25 colleagues who work in the office of academic affairs at hospitals all over China. They helped us send the link to qualified physicians and researchers they have access to, mainly through WeChat, a popular social media app in China. In addition, some physicians getting knowledge of the study also helped with distributing the questionnaire to qualified physicians or researchers, making it difficult to calculate the total number of people who have received the link. Participants could fill the questionnaire by clicking the QR Code. The participants were informed that confidentiality couldn’t be guaranteed in this study. Information on their experience in clinical research and their needs for clinical research training was collected. The number of those who received the link but didn’t fill the questionnaire could not be known.

## Results

The respondents provided related information about their knowledge of and participation in clinical research, the difficulties they encountered in practice and what they expected from clinical research training.

From June 11 2020 to June 18 2020, we received 605 completed questionnaires, of which 600 were valid. The average time to finish the questionnaire was about 5 min. 600 participants come from 28 provinces, autonomous regions and municipalities in China, included 507 clinicians, 58 full-time clinical researchers and 35 full-time basic medical researchers as shown in Table 1.

**Table 1:** Demographics of participants

Item	Characteristics	Number (%)
Age	31-40	299(49.8 %)
	Under 30	162(27.0 %)
	41-50	111(18.5 %)
	Over 50	28(4.7 %)
Location	East China	378(63.0 %)
	South China	66(11.0 %)
	North China	63(10.5 %)
	Northeast China	37(6.2 %)
	Central China	32(5.3 %)
	Southwest China	24(4.0 %)
Discipline	Internal Medicine	310(56.7 %)
	Surgery	124(23.5 %)
	Medical Technology	39(7.2 %)
	Pediatrics	13(2.2 %)
	Obstetrics and Gynecology	12(2.0 %)
	Others (pharmacy, epidemiology, etc.)	102(6.9 %)
Position	Clinicians	207(84.5 %)
	Full-time clinical researcher	58(9.7 %)
	Full-time basic medical researcher	35(5.8 %)
Types of hospital the respondent works for	Top hospital (Tertiary hospital)	555(92.5 %)
	Second-class hospital	22(3.7 %)
	Others (private hospital, etc.)	23(3.8 %)

### Current research practice

Current research practice was evaluated from three perspectives, i.e., types of clinical research they participated in, types of work undertaken and difficulties encountered during research. Results showed that 125 (20.8 %) participants had led or participated in all types of clinical studies, and 84 (14 %) had not participated in any listed type of clinical studies. 36 (6 %) participants had frequently involved in all types of work during clinical studies. More information on participation and work undertaken in clinical studies is shown in Table 2.

**Table 2:** Participation status and work undertaken in clinical studies

Item			Particulars			
**Types of clinical study**		**Leader** **N (%)**		**Investigator** **N (%)**	**Never** **N (%)**	
Multi-center randomized controlled trials		17(2.8 %)		291(48.5 %)	292(48.7 %)	
Single-center randomized controlled trials		53(8.8 %)		279(46.5 %)	268(44.7 %)	
Prospective cohort studies		35(5.8 %)		239(39.8 %)	326(54.4 %)	
Retrospective cohort studies		87(14.5 %)		290(48.3 %)	223(37.2 %)	
Case-control studies		83(13.8 %)		275(45.8 %)	242(40.4 %)	
Cross-sectional studies		57(9.5 %)		189(31.3 %)	354(59.0 %)	
Case reports		117(19.5 %)		261(43.5 %)	222(37.0 %)	
**Types of work undertaken**		**Frequently** **N (%)**		**Rarely** **N (%)**	**Never** **N (%)**	
Clinical trial design		166(27.7 %)		318(53.0 %)	166(19.3 %)	
Clinical trial protocol writing		15(25.0 %)		319(53.2 %)	131(21.8 %)	
Biological sample collection		223(37.2 %)		261(43.5 %)	116(19.3 %)	
Biobank management		112(18.7 %)		290(48.3 %)	198(33.0 %)	
Database maintenance		128(21.3 %)		290(48.3 %)	182(30.4 %)	
Cohort follow-up		161(26.8 %)		288(48.0 %)	151(25.2 %)	

Difficulties encountered during clinical research and subgroup analysis by age are shown in Table 3. More than 70 % of the participants reported difficulties in data statistics, and over 50 % reported difficulties in cohort and biobank construction, data management and clinical study design. More than 40 % of the participants found it difficult to propose a proper scientific research problem.

Results of subgroup analysis indicate that as age increases, even with more accumulation of clinical experience, some difficulties still persist, such as “data statistics”, “cohort and biobank construction”, “data management” and “insufficient funding for study”. However, the proportion of difficulties such as “lack of knowledge of study design and operation”, “difficulty in follow-up” appear to be on a downward slope as the participants get older.

**Table 3:** Difficulties encountered in clinical research

Items	In total N(%)	Subgroup analysis			
		**Under 30** ** N (%)**	**31-40** ** N (%)**	**41-50** ** N (%)**	**Over 50** ** N (%)**
Data statistics	425(70.8 %)	90(55.6 %)	225(75.3 %)	92(82.9 %)	18(64.3 %)
Cohort and biobank construction	356(59.3 %)	84(51.9 %)	188(62.9 %)	66(59.5 %)	18(64.3 %)
Data management	355(59.2 %)	76(46.9 %)	190(63.6 %)	76(68.8 %)	13(46.4 %)
Lack of knowledge of study design and operation	310(51.7 %)	84(51.9 %)	163(54.5 %)	54(48.7 %)	9(32.1 %)
Insufficient funding for study	291(49.2 %)	67(41.4 %)	150(50.2 %)	59(53.2 %)	15(53.8 %)
Difficulty in follow-up	295(48.5 %)	89(55.0 %)	143(47.8 %)	52(46.9 %)	11(39.3 %)
Insufficient clinical cases	264(44.0 %)	73(45.1 %)	132(44.2 %)	50(45.1 %)	9(32.1 %)
Difficulty in proposing research questions	256(42.7 %)	162(48.2 %)	136(45.5 %)	37(33.3 %)	5(17.9 %)

#### The training needs for clinical research

 The training needs for clinical research fall into the following categories: data statistics (application of statistical software, principles and methods of statistics, data in-depth analysis and interpretation), study design (clinical study design, questionnaire design), cohort and biobank construction and maintenance, publication, study ethics and regulation. Through clinical research training, participants expected to have access to resources for clinical research, capacity enhancement, opportunities to participate in clinical research, publication and training certification. Detailed results are shown inTable 4.

**Table 4:** Training needs and expectations

Items			N(%)
Needs			
Data statistics			
Application of statistical software			395(65.8 %)
Principles and methods of statistics			356(59.3 %)
In-depth analysis and interpretation of data			243(40.5 %)
Study design			
Clinical trial design			342(57.0 %)
Questionnaire design			119(19.8 %)
Cohort and biobank construction and maintenance			
Cohort and database construction			266(44.3 %)
Establishment and maintenance of biobank			197(32.8 %)
Publication			
Paper writing and publication			261(43.5 %)
Research ethics and regulation			
Clinical research ethics and regulation			156(26.0 %)
Expectations			
Resources for clinical research			
Financial support for study			300(50.0 %)
Access to certain clinical research resources			291(48.5 %)
Capacity enhancement			
Improving diagnosis and treatment capabilities			295(49.2 %)
Opportunity to participate in clinical research			
Participation in clinical research projects			214(35.7 %)
Conducting study design under guidance			178(29.7 %)
Establishing a one-on-one mentoring relationship with instructors			164(24.0 %)
Publication			
Publication of clinical research papers			455(75.8 %)
Training certification			
Earning continuing education credits			41(6.8 %)
Receiving a training certificate			38(6.3 %)

## Discussion

Most of the respondents (92.5 %) work at top-level hospitals (also named tertiary hospital) and most of them (85 %) have led or participated in various types of clinical research. However, even physicians from top-level hospitals are faced with varied types of difficulties during clinical research, though many of them have rich experience in this field. On the one hand, many clinicians in China are preoccupied with routine clinical practice, leaving little time to conduct clinical research. On the other hand, many physicians feel the pressure to publish papers for promotion. Therefore, most of them often undertake only one type instead of varied types of work during research projects without understanding the whole process of the research.

Physicians of different ages appear to have different problems during clinical research. Those over 30 years old, for example, are more likely to have problems with medical statistical analysis than those under 30, which may be accounted for by the fact that the younger generation of physicians have more opportunities for earlier training on this front than their senior counterparts. Those aged between 41 and 50, the eldest group among the respondents, are least likely to have problems with understanding the design and conduction of clinical research, which is probably attributed to the accumulation of experience. Given such differences, training programs on clinical research should be customized with different priorities for different age groups.

While the most common problem is with data statistical analysis, other types of problems also occur at high frequency, indicating a strong demand from Chinese physicians for systematic clinical research training. Besides medical statistical analysis, all age groups also have problems with cohort and biobank construction and data management. It is expected that many of these problems can be addressed by providing training through dedicated programs, which will in turn enhance their research skills, sharpen their scientific acumen, and ultimately, improve the quality of clinical research.

In addition to systematic training, we believe that establishing rounded scientific team is also a very important aspect to solve the above-mentioned problems. Making every physician to become a talented statistician is not the key solve the problem of “data statistics”. So expect for specific research principle and method raining, to help physician get prepared to be a qualified “team leader”, who is able to gain proper manpower, materials and finances and manage whole process of clinical research, is also an indispensable perspective of training.

In this study, a high percentage of all age groups reported limited funding as a problem for conducting clinical research, suggesting that a training program may be more productive if it can get physicians get more prepared for funding application or even provide more access to potential sources of research funding. Most of hospitals in China provide start-up funds for physicians and researchers in their early career stages. It could be better if research funds can be integrated with clinical training, which would require systematic arrangements. China’s investment in clinical research will continue to rise. Meanwhile, targeted training programs based on the unmet needs are required to ensure that these resources are put to good use.

“How to support physician-scientist” has been listed as one of the seven major challenges posed to global health by the World Health Organization in the next decade [16]. More and more emerging types of clinical research, for example real-world study, force great challenges for physicians. Systematic training of different types of clinical research, comprising of research question proposal, study protocol formulation, study process control, data management and analysis, transformation of clinical research achievements, medical professional education and patient education, is in urgent need [17–20]. In addition, physicians should pay more attention that it is important to transform clinical research input into effective clinical output, so as to truly achieve the value of clinical research [21, 22].

This exploratory study indicated that there may be huge needs for clinical research training in China, yet it is hard and costly to design and carry out dedicated training programs. Fortunately, the imbalance between supply and demand of clinical research training has been increasingly recognized, more and more clinical research training is launched by top-level hospitals and medical education institutions.

This study has several limitations. First, cross-sectional data were obtained using convenience sampling, making it weak to generalize the findings to physicians at other less prestigious hospitals. Second, the analysis in this study is solely based on self-reported data which might be subject to social desirability bias, leading to overestimated mastery of clinical research knowledge. Third, most of the respondents (over 60 %) were from east China, making it less representative of clinical research training needs in other regions of China. Fourth, this study has its focus on biomedical, positivist types of research over other types.

## Conclusions

The study showed that even physicians in top hospitals tend to encounter various difficulties when conducting clinical research in China, such as data statistics, cohort and biobank construction and data management. This study highlights the importance of systematic training for future physician scientists in China. A clinical research training program developed by the author team is underway, and we will report the training program in the future.

## Supplementary Information

**Additional file 1.**

## Data Availability

The datasets used during the current study are available from the corresponding author on reasonable request.
