# Supplementary material for: An exploratory study on needs for clinical research training: data from Chinese hospitals
Source: BMC Med Educ. 2021 Nov 2;21:559. doi: 10.1186/s12909-021-02993-1 (PMC8564980; doi:10.1186/s12909-021-02993-1)
Supplement: Supplementary file 1 — Additional file 1. [file 12909_2021_2993_MOESM1_ESM.docx]

**Questionnaire in the survey**

| 1.Age | - Under 30 - 31-40 - 41-50 - Above 50 |
| --- | --- |
| 2.Major | - Internal Medicine - Surgery - Medical Technology - Pediatrics - Obstetrics and Gynecology - Others (pharmacy, epidemiology, etc.) |
| 3.Academic degree | - Ph.D. - Master - Graduate |
| 4.Are you applying for another academic degree now? | - Yes - No |
| 5.Occupation | - Clinicians - Full-time clinical researcher - Full-time basic researcher |
| 6.Job title | - Senior - Vice senior - Intermediate - Junior |
| 7.Type of the hospital you are working for | - Top-level hospital (Tertiary hospital) - Secondary hospital - Others (private hospital, etc.) |
| 8.Location | - North China - East China - Central China - South China - Southwest China - Northeast China |
| 9.How well do you think you know about clinical medical research? | - Mastered the common clinical medical research design and statistical methods - Only understood the basic concepts of clinical medical research - Did not understand the related contents of clinical medical research |
| 10.How often do you take part in such work in clinical research? | - Cohort follow-up - Biological sample collection - Biobank management - Database maintenance - Clinical study data analysis - Clinical trial design - Clinical trial protocol writing |
| 11.Can you describe the difficulties you encountered in clinical research? | - Medical statistical analysis problem - Cohort and biospecimen repository creation problem - Data management problem - Lack of understanding of how clinical studies are designed and conducted - Limited funding for studies - Difficulty with follow-up - Limited number of clinical cases - Not aware of topics worthy of study in this specialty |
| 12.Which type of clinical research have you led or took part in? | - Multi-center randomized controlled trials - Single-center randomized controlled trials - Prospective cohort studies - Retrospective cohort studies - Case-control studies - Cross-sectional studies - Case reports |
| 13.Have you ever published research paper in English? | - Yes - No |
| 14.Have you ever published paper of clinical research in English as first author or corresponding author? | - Yes - No |
| 15. Have you ever published clinical research paper in English? | - Yes - No |
| 16.Have you ever took part in clinical research training? | - Yes - No |
| 17.What organization provided clinical research training for you? | - Self-learning - Training organized by the hospital - Undergraduate and graduate school courses - Training organized by other institutions outside the hospital |
| 18.What clinical research knowledge or technology are you interested in? | - Common statistical analysis and use of statistical software - Fundamentals of medical statistics/biostatistics - Clinical trial design - Cohort creation and corresponding database creation and maintenance - Paper writing and publication - Secondary analysis of data - Establishment and maintenance of biospecimen repositories - Clinical research ethics and regulation - Drug clinical trials - Questionnaire design |
| 19.What clinical research knowledge are you eager to learn currently? | - Common statistical analysis and use of statistical software - Fundamentals of medical statistics/biostatistics - Clinical trial design - Cohort creation and corresponding database creation and maintenance - Paper writing and publication - Secondary analysis of data - Establishment and maintenance of biospecimen repositories - Clinical research ethics and regulation - Drug clinical trials - Questionnaire design |
| 20.Which kind of clinical research training do you prefer? | - Online lectures mainly, supplemented by face-to-face lectures - Face-to-face lectures mainly, supplemented by online lectures - All online classes - All face-to-face |
| 21.Do you think there should be examine for the trainees to evaluation the effect of clinical research training? | - Necessary - No need for that - No idea |
| 22.Which incentive could encourage you to take part in clinical research training? | - Publication of clinical research papers - Receiving clinical research funding support - Participation in specialized disease alliances to develop specialized disease diagnosis and treatment capabilities - Access to certain clinical research case analysis resources - Participation in a clinical research project as part of a multi-center unit - Working with a lecturer on a project design - Establishing a one-on-one mentoring relationship with an instructor of the same professional background - Earning continuing education credits - Receiving a training certificate |
| 23.What characteristics or qualification do you think the trainers of clinical research should have? | - Training experience overseas - Have published research paper in NEJM, JAMA, Lancet or BMJ as corresponding author - Same academic background with trainees - Senior title - Fellowship in Chinese Medical Association or other organization - No specific requirement |
|  |  |
